# The Atlanta Medical Society

**Published:** 1882-10

**Authors:** 


### Societies.

---

### THE ATLANTA MEDICAL SOCIETY.

*Reported for the Atlanta Medical Register.*

Atlanta, August, 1882.

Dr. V. H. Taliaferro in the chair.

Dr. W. S. Elkin, the essayist for the evening, read a paper on Experimental and microscopical researches in traumatic injuries of the liver, and hepatic abscess. See page 1.

After the discussion of this paper, which was participated in by several members, under the call for voluntary reports:

Dr. Jas. B. Baird reported a case of fracture of the clavicle about the junction of the outer and middle third, in a boy aged fourteen years, the result of a fall on the point of the shoulder, backwards from a wagon.

The case presented nothing peculiar, and he only referred to it for the purpose of testifying to the comfort which the patient derived from the application of a simple and well-known dressing, which consisted merely of two strips of adhesive plaster about three inches wide. One was looped around the arm of the injured side, about the insertion of the deltoid muscle, and drawing the arm sharply backwards, was made to encircle the chest, and, to insure firm adhesion, was caused to overlap on the back. The second strip was attached to the point of the shoulder on the sound side and carried diagonally across the back, under the elbow of the affected side, drawing the arm firmly forward—the first strip acting as the fulcrum, the second as the power—and passed along the outer border of the forearm and over the dorsum of the hand—which should be laid on the pectoral region of the sound side, the forearm being in a line with the course of the adhesive strip—and finally attached to the other end of the strip, at the point of departure, on the shoulder opposite the injury. No axillary pad was used, but a wad of cotton was placed between the elbow and the strip of plaster to prevent excoriation. A few stitches, here and there, at places where the strips unite or cross each other will materially strengthen the dressing.

Dr. F. H. O'Brien had recently treated a girl, aged twelve years in the manner described by Dr. Baird, for fractured clavicle. It is the most popular and satisfactory method of dealing with such injuries. In his case no deformity resulted. The temporary callous soon disappeared. Used at first an axillary pad, but it proved uncomfortable and it was discarded, after which the case progressed with entire satisfaction.

Dr. O'Brien also reported an amputation of the foot for necrosis of the astragalus, the result of a dislocation. The amputation was performed at the junction of the lower and middle third of the leg. It yielded him an interesting pathological specimen, which he will take occasion to exhibit to the society.

